# 

**Published:** 1900-12

**Authors:** 


### CONTENTS.

ORIGINAL COMMUNICATIONS. PAGE

Some German Literature of Interest to the Profession. By WILLIAM H. POTTER, D.M.D., Boston, Mass. . . . . 781
State Dental Examining Boards and their Questions. By WILLIAM H. TRUEMAN, D.D.S., Philadelphia . . . . . 784
Prophylaxis in Dentistry. By D. D. SMITH, D.D.S., M.D. . . . . 805
What does it mean? By DR. B. F. ARRINGTON, Goldsboro, N. C. . . . . 811

REPORTS OF SOCIETY MEETINGS.

American Academy of Dental Science . . . . . 814

EDITORIAL.

The Last Month of the Century . . . . . 818
What were the Motives? . . . . . 821
The Galveston Sacrifice . . . . . 823
Dr. Clapp's Reply . . . . . 823
Charges made by the New Jersey State Board . . . . . 824
Correction . . . . . 824

BIBLIOGRAPHY . . . . . 825

DOMESTIC CORRESPONDENCE.

Dr. Clapp's Rejoinder . . . . . 831

FOREIGN CORRESPONDENCE.

Reply to Dr. Hopkins . . . . . 843

NOTES AND COMMENTS . . . . . 844

CURRENT NEWS.

Board of Examiners of Pennsylvania . . . . . 845
The Trouble in the New Jersey State Board . . . . . 845
Central Dental Association of Northern New Jersey—Resolutions of Confidence in Dr. Meeker . . . . . 849
American Dental Society of Europe . . . . . 850
Institute of Dental Pedagogics . . . . . 850

